# Subcutaneous Granulomatous Inflammation due to Basidiobolomycosis: Case Reports of 3 Patients in Buruli Ulcer Endemic Areas in Benin

**DOI:** 10.1155/2018/1351694

**Published:** 2018-01-10

**Authors:** Luc V. C. Brun, Jean Jacques Roux, Ghislain E. Sopoh, Julia Aguiar, Miriam Eddyani, Wayne M. Meyers, Dirk Stubbe, Marie T. Akele Akpo, Françoise Portaels, Bouke C. de Jong

**Affiliations:** ^1^Department of Pathology, School of Medicine, University of Parakou, 03 BP 333 Parakou, Benin; ^2^Department of Pathology, Hospital of Chambéry, Place Lucien Biset, 73000 Chambéry, France; ^3^Buruli Ulcer Treatment Center, Allada, Benin; ^4^Regional Institute of Public Health, Ouidah, Benin; ^5^Nutritional Center of Gbemontin, Zagnanado, Benin; ^6^Institute of Tropical Medicine, Nationalestraat 155, 2000 Antwerpen, Belgium; ^7^Armed Forces Institute of Pathology, Washington, DC, USA; ^8^BCCM/IHEM Biomedical Fungi and Yeasts Collection, Scientific Institute of Public Health, Brussels, Belgium; ^9^Department of Pathology, School of Medicine, University of Abomey Calavi, Cotonou, Benin

## Abstract

**Background:**

Basidiobolomycosis is a rare subcutaneous mycosis, which can be mistaken for several other diseases, such as soft tissue tumors, lymphoma, or Buruli ulcer in the preulcerative stage. Microbiological confirmation by PCR for* Basidiobolus ranarum* and culture yield the most specific diagnosis, yet they are not widely available in endemic areas and with varying sensitivity. A combination of histopathological findings, namely, granulomatous inflammation with giant cells, septate hyphal fragments, and the Splendore-Hoeppli phenomenon, can confirm basidiobolomycosis in patients presenting with painless, hard induration of soft tissue.

**Case Presentations:**

We report on three patients misdiagnosed as suffering from Buruli ulcer, who did not respond to Buruli treatment. Histopathological review of the tissue sections from these patients suggests basidiobolomycosis. All patients had been lost to follow-up, and none received antifungal therapy. On visiting the patients at their homes, two were reported to have died of unknown causes. The third patient was found alive and well and had experienced local spontaneous healing.

**Conclusion:**

Basidiobolomycosis is a rare subcutaneous fungal disease mimicking preulcerative Buruli ulcer. We stress the importance of the early recognition by clinicians and pathologists of this treatable disease, so patients can timely receive antifungal therapy.

## 1. Introduction

Basidiobolomycosis is a subcutaneous mycosis that occurs in tropical and subtropical regions of Asia, Africa, and South America, caused by* Basidiobolus ranarum *Eidam or related species [1]. The disease mainly affects children and tends to preferentially affect the thighs, buttocks, or trunks. The clinical and histopathological signs at first do not evoke a fungal disease. The initial infection is subacute, sometimes marked by a pseudophlegmon or erysipelas. The evolution is slow and gradual, with periods of remissions over several months or years, in the form of an extensive plaque. Patients on immunosuppressive therapy may be at higher risk of developing progressive disease [2]. Clinical examination reveals inhomogeneous swellings. Palpation reveals irregular firm nodules. The edema is nonpitting and hard as wood, and the skin is rigid and cannot wrinkle. The symptomatology varies, depending on the evolutionary stage (early versus chronic infection). In early infection, nodules are hot and slightly painful. In chronic infection, they are cold and painless. This soft tissue infection usually respects the joints and muscles, infrequently causing functional impairment [3].

Although medical treatment by oral potassium iodide and ketoconazole can be effective [4], the untreated infection can be fatal. Over the past 40 years, 179 cases of subcutaneous basidiobolomycosis have been reported worldwide [5], including West Africa [6–10]. Among them, there were only seven cases, recently reported in Benin [10].

*Basidiobolus ranarum *belongs to the fungal phylum Entomophthoromycota. This phylum holds over 250 species, mostly soil or litter saprophytes, but also numerous arthropod pathogens. It has emerged as one of the major lineages of the dissolved phylum Zygomycota (which, at the time, also included the now independent subphylum Mucoromycotina). Within the phylum Entomophthoromycota, two species are known as human pathogens:* Conidiobolus coronatus* (Costantin) Batko (in the order Entomophthorales, causing rhinophycomycosis) and* B. ranarum* (in the order Basidiobolales). The genus* Basidiobolus *contains at least six species [11]. In addition to* B. ranarum*, also* B. meristosporus *Drechsler and* B. haptosporus *Drechsler have been identified as causal agents of basidiobolomycosis. These latter two names are currently accepted as synonyms of* B. ranarum*. However, this is not corroborated by the multigene analysis of Gryganskyi [11] and more taxonomic research will be needed to resolve this delineation of the taxonomy.* Basidiobolus *spp. have been isolated from reptile or amphibian dung, but also bats and other (insectivorous) animals have been identified as carriers and possible reservoirs [12–15].

The transmission route of* Basidiobolus *spp. is poorly understood. Basidiobolomycosis develops in the subcutaneous soft tissue and yet can involve muscles and surrounding organs, as well as gastrointestinal lesions [16, 17].

Basidiobolomycosis can be mistaken for Buruli ulcer (BU), an infectious skin disease due to* Mycobacterium ulcerans*, also endemic in some countries in Africa, Asia, South America, Mexico, and Australia, which presents as a painless papule, nodule, plaque, edema, or ulcer. The infrequent encounter of basidiobolomycosis and its symptoms that can mimic a BU plaque or oedematous form limits clinician experience in its recognition in the differential diagnosis. Histopathological examination of a tissue biopsy can provide diagnostic clues, when a dermohypodermic granuloma is observed, rich in eosinophils and giant cells, with the presence of hyphae. The phenomenon of Splendore-Hoeppli consists of an asteroid body, corresponding to eosinophilic material often in a stellate or club form [18], organized around various microorganisms such as bacteria, fungi, parasites, or even biologically inert substances [18], yet mostly associated with basidiobolomycosis [4, 19]. Microbiological confirmation of the diagnosis of basidiobolomycosis is based on the detection of colonies of* B. ranarum* after seeding a biopsy specimen on Sabouraud medium [20] or PCR detection of specific targets. We describe three patients with subcutaneous basidiobolomycosis in a Buruli Treatment Center (CDTUB) in Benin, two of whom had died on follow-up.

## 2. Case Presentation

### 2.1. Patient 1

A malnourished three-year-old girl living in a BU-endemic area was admitted to the CDTUB of Zagnanado in 1997 for a two-month history of swelling of the right flank. The lesion was a hard painless plaque. Based on the clinical signs, in conjunction with residence in a BU-endemic area, an initial diagnosis of BU was made. Surgical excision was performed and samples were collected for bacteriology,* M. ulcerans* specific PCR, and histopathology. Treatment with cloxacillin and gentamycin was instituted. Direct microscopy of the tissue samples after Ziehl-Neelsen staining was negative for acid fast bacilli (AFB). The IS*2404* PCR for* M. ulcerans* was also negative. However the histopathological examination of the tissue samples after Hematoxylin-Eosin (HE) and Gomori- Methenamine-Silver (GMS) staining provided strong support for the diagnosis of basidiobolomycosis. PCR for the detection of* B. ranarum* DNA was not performed. No information was available about the disease progression in this patient at the time she was lost to follow-up. However, she was recently reidentified and had undergone spontaneous healing.

### 2.2. Patient 2

A 43-year-old man living in a rural BU-endemic area presented in the CDTUB of Zagnanado in 2009 with an extensive atypical cutaneous plaque on the right thigh that had developed over six months. He received traditional herbal treatment before he presented. On palpation, the lesion was painless with very hard induration, and the patient was afebrile. Based on the clinical signs and symptoms, as well as residence in a BU-endemic region, an initial diagnosis of BU was made. Surgical excision was performed and tissue samples were collected for laboratory confirmation of BU. Laboratory tests (direct smear examination after Ziehl-Neelsen staining, culture, and IS*2404* PCR) were negative.

Histopathological examinations revealed a granulomatous inflammation with giant cells without caseating necrosis, reported as tuberculoïd granulomas. The patient had received antibiotic treatment for BU (streptomycin and rifampicin for 8 weeks) and cloxacillin, without improvement.

Subsequently, the lesion extended towards the right leg and buttock. The patient was lost to follow-up. His relatives mentioned that the lesions progressed from nonulcerated lesions to an ulcerated one. He died of unknown causes.

Five years after the patient's death, histopathological reexamination suggested the diagnosis of basidiobolomycosis based on the presence of eosinophilic and giant multinucleated cells mixed with septate hyphal fragments and the Splendore-Hoeppli phenomenon. We submitted frozen tissue samples for PCR at the BCCM/IHEM culture collection for biomedical fungi at the Scientific Institute of Public Health in Belgium, but these were negative for* B. ranarum*.

### 2.3. Patient 3

**Figure 1:**
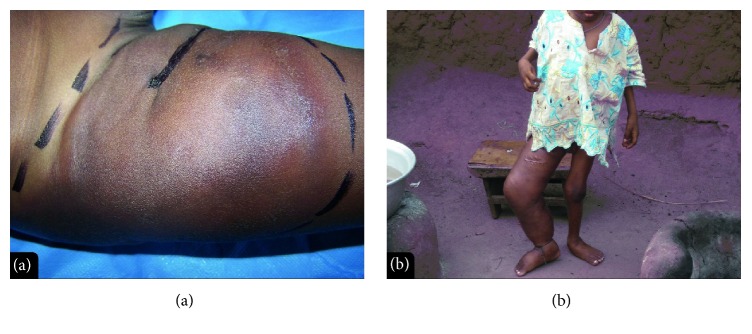
Clinical aspects (patient 3). (a) Lesion on R thigh, recurrence after initial resection. (b) The same patient three years later (a few months before his death).

A three-year-old boy living in a rural BU-endemic area was admitted in the CDTUB of Zagnanado in 2012, for an atypical cutaneous and extensive plaque-like lesion. The lesion was located on the right thigh (Figure 1(a)) and clinically resembled BU or a soft tissue tumor. The lesion was painless, had the hardness of wood, and started one month before admission. The patient was in good overall physical condition. Laboratory tests on biopsy samples for BU (direct smear examination after Ziehl-Neelsen staining, culture, and IS*2404* PCR) were negative.

The initial histopathological examination was interpreted as “possible BU” because of the observed granulomatous inflammation. Despite BU specific antibiotic treatment (streptomycin and rifampicin for 8 weeks) and cloxacillin, as well as surgical excision of the lesion, the induration recurred before the surgical wound had healed. The lesion then extended towards the leg and buttock. The patient was lost to follow-up, despite attempts to visit him at home, and had been taken to a traditional practitioner. When the histopathological diagnosis was revised as suggesting basidiobolomycosis rather than BU, the parents refused free treatment with ketoconazole. The lesion extended to the whole right lower limb with a pseudoelephantiasis aspect (Figure 1(b)), and the skin ulcerated on the calf. The patient died two years after the onset of the illness. A PCR for* B. ranarum,* performed at BCCM/IHEM on DNA extracted from frozen tissue suspensions, was negative.

Histopathological features were consistent with basidiobolomycosis, based on the presence of eosinophilic cells and giant multinucleated cells mixed with rare septate hyphal fragments and Splendore-Hoeppli material.

The families of all three patients provided written informed consent for these anonymized case reports despite refusal of free treatment (patient 3).

### 2.4. Pathology Results

**Figure 2:**
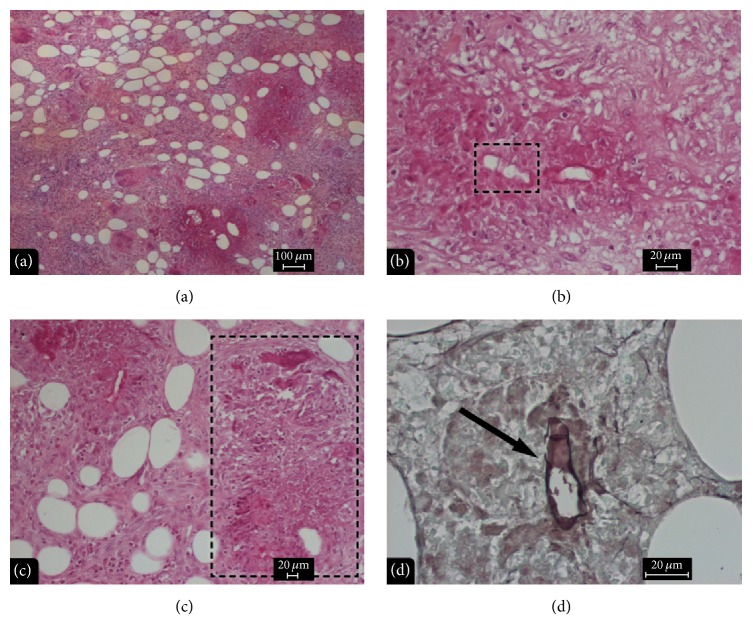
Histopathology of basidiobolomycosis. (a) Granulomatous inflammation with eosinophils and multinucleated giant cells in the subcutaneous tissue. Hematoxylin-Eosin, 50x. (b) Granulomatous inflammation with eosinophils cells and septate hyphal fragments. Hematoxylin-Eosin, 100x. (c) Granulomatous inflammation with eosinophils, multinucleated giant cells, and the Splendore-Hoeppli phenomenon. Hematoxylin-Eosin, 100x. (d) Septate hyphal fragments (arrow). Gomori-Grocott staining, 400x.

Histopathological results after HE staining of all three cases showed tuberculoïd granulomata with giant cells (Figure 2(a)), numerous lymphocytes, histiocytes and eosinophilic cells (Figure 2(b)), an amorphous eosinophilic material also known as the “Splendore-Hoeppli phenomenon” (Figure 2(c)), and 10 *μ*m diameter septate hyphal fragments (Figure 2(b)), confirmed also by the Gomori-Grocott staining (Figure 2(d)). These hyphal fragments sometimes appeared centered within a Splendore-Hoeppli phenomenon material or were found in the cytoplasm of multinucleated giant cells.

## 3. Discussion

In these three patients the diagnosis of basidiobolomycosis was histologically suggested, based on the presence of a very hard panniculitis with a significant scleroinflammatory response (lymphocytes, histiocytes, eosinophiles, and giant cells), few septate hyphae, and the presence of the Splendore-Hoeppli phenomenon. PCR was negative for* B. ranarum*. The differential diagnosis provided for basidiobolomycosis includes soft tissue tumors, such as a synovial sarcoma [21], Hodgkin lymphoma [22], and mycetoma and BU [5]. Indeed, a tuberculoïd granuloma with giant cells can also be observed in BU lesions, especially on healing. However, neither the septate hyphal fragments nor the Splendore-Hoeppli phenomenon is observed in BU lesions.

Importantly, BU differs from basidiobolomycosis in etiology and management. The Splendore-Hoeppli phenomenon itself is not specific for basidiobolomycosis [18] and can also be observed in other infections, such as botryomycosis [23] bronchocentric granulomatosis due to* Aspergillus* [24] mycetoma [25] and* Pityrosporum *folliculitis skin infection [26], although the combination of the clinical presentation, the Splendore-Hoeppli phenomenon, and septate hyphae suggests basidiobolomycosis. In addition to a predilection for lungs rather than the subcutis,* Aspergillus* can be recognized by septate hyphae that branch at 45° angles and by vascular invasion, while mycetoma produces grains and has a very distinct clinical presentation from basidiobolomycosis. Since culture of the offending fungus* B. ranarum* is difficult [3], clinical and histopathological features can help to suggest the diagnosis of basidiobolomycosis. Among seven cases recently reported in Benin by dermatologists, five whose tissues were sampled by biopsy were diagnosed using histopathology [10]. Early recognition of basidiobolomycosis would allow early effective treatment, even in the absence of culture or PCR positive results. The detection of fungal pathogens by PCR is particularly challenging. Fungal cell walls are not easily lysed for the release of DNA, which leads to false-negative PCR results [27]. Isolation of* Basidiobolus ranarum* DNA from archival formalin-fixed, paraffin embedded (FFPE) tissue blocks has been reported, with a protocol allowing reliable purification of fungal DNA [28, 29]. The antifungals that have most been used for treatment of basidiobolomycosis are oral itraconazole [10, 30, 31] and oral ketoconazole at a dose of 400 mg per os once daily [32] or 7 to 10 mg per kilogram once daily (for children), for two to six months [6, 7, 10, 33]; posaconazole has also been effectively used in one case report [34]. Potassium iodide may be another effective treatment option [35].

None of the patients in our series benefited from ketoconazole treatment. The third patient's parents refused this outpatient treatment, and his disease progressed, causing elephantiasis-like lymphedema (Figure 1(b)), as described by Kamalam and Thambiah [36]. We hypothesize that the parents' refusal stemmed from a loss of confidence in allopathic medicine due to the delayed diagnosis of basidiobolomycosis, which was preceded by erroneous treatment for BU including surgical excision. With the failure of allopathic medical and surgical therapies the parents interpreted the unfavorable evolution as evidence for witchcraft (enchantment); in Benin this culturally supported framework for understanding disease warrants traditional therapies including herbal treatments, rather than allopathic approaches [37–40]. It is important that health workers, especially in BU-endemic areas, recognize the clinical symptoms of basidiobolomycosis and ask for histological testing in order to avoid delays in diagnosis and appropriate treatment for this potentially devastating disease.

## 4. Conclusion

Clinicians need to recognize wood-like induration of skin and soft tissue as a potential deep-seated fungal infection, also in BU-endemic areas. Such patients first and foremost need antifungal treatment, preferably after a biopsy is taken for histopathological features characteristic for basidiobolomycosis, rather than a wide excision.

Failure to recognize this clinical entity may result in therapeutic failure and may have contributed to the death of two of these patients. Clinical instruction on this presentation is therefore of paramount importance in tropical healthcare education.
